# Comparison of lenvatinib plus pembrolizumab versus first-line systemic chemotherapy for advanced intrahepatic cholangiocarcinoma: a real-world retrospective study

**DOI:** 10.3389/fimmu.2024.1494520

**Published:** 2024-11-29

**Authors:** Zhenyun Yang, Weijie Wu, Zhiwen Hu, Yizhen Fu, Zili Hu, Yangxun Pan, Juncheng Wang, Jinbin Chen, Zhongguo Zhou, Yaojun Zhang, Minshan Chen, Dandan Hu

**Affiliations:** ^1^ Department of Liver Surgery, Sun Yat-sen University Cancer Center, Guangzhou, China; ^2^ Collaborative Innovation Center for Cancer Medicine, State Key Laboratory of Oncology in South China, Sun Yat-Sen University Cancer Center, Guangzhou, China; ^3^ Guangdong Provincial Clinical Research Center for Cancer, Sun Yat-Sen University Cancer Center, Guangzhou, China

**Keywords:** intrahepatic cholangiocarcinoma, lenvatinib, pembrolizumab, systemic chemotherapy, survival, adverse events

## Abstract

**Background:**

Systemic chemotherapy (SC) stands the only first-line treatment for advanced intrahepatic cholangiocarcinoma (iCCA) for the past few decades. Immune checkpoint inhibitors (ICIs) have been proved to provide additional benefit in disease control. However, oncological outcome of iCCA remains poor and awaits further improvement with new treatment modalities. Promising results have been observed in lenvatinib plus pembrolizumab (Len-P) as a second-line therapy in iCCA. This study aimed to explore the safety and efficacy of Len-P as a first-line therapy for iCCA patients in real-world clinical practice.

**Methods:**

We retrospectively enrolled 133 patients with advanced iCCA who received Len-P or SC between May 2019 and May 2023. Overall survival (OS), progression-free survival (PFS), objective response rate (ORR), disease control rate (DCR), and adverse events (AEs) were compared between the two groups.

**Results:**

There were 72 patients and 61 patients in the Len-P and SC groups, respectively. The median OS for the Len-P and SC groups was 16.3 and 17.8 months, respectively. The median PFS for the Len-P and SC groups was 8.9 and 11.4 months, respectively. There was no significant difference in ORR and DCR between the Len-P and SC groups (ORR: 22.2% vs. 23%; P=0.92; DCR: 69.4% vs. 77%; P=0.58). Additionally, the overall incidence of AEs was lower in the Len-P group than SC group. Low inflammation-based scores were indicative of favorable outcomes in patients undergoing Len-P therapy.

**Conclusion:**

This study demonstrated that Len-P is promising for the treatment of advanced ICC, with highly improved safety. It emerges as a viable treatment alternative for advanced iCCA. Inflammation-based scores show potential utility in identifying individuals likely to benefit from Len-P therapy.

## Introduction

Intrahepatic cholangiocarcinoma (iCCA), the second most common primary liver cancer, is characterized by surreptitious presentation, aggressive progression, and unfavorable prognosis (1–3). Recent data indicates a concerning rise in the annual incidence rate of iCCA in China, with an average annual increase of 11.1% (4). Complete surgical resection is currently the only potential curative treatment for iCCA, however, approximately 70-80% of patients are deemed tumor unresectable due to an advanced stage at diagnosis (5). Thus, there is a critical need for effective systemic treatments.

Currently, the first-line chemotherapy regimen for patients with advanced biliary tract cancers (BTCs) is gemcitabine with cisplatin (GEMCIS), which has a modest median overall survival (OS) of 11.7 months and median progression-free survival (PFS) of 8.0 months (6). Similarly, gemcitabine with oxaliplatin (GEMOX) manifests comparable efficacy for the treatment of BTCs (7, 8). However, gemcitabine-based chemotherapy is associated with a high incidence of adverse events, especially hematological toxicities, significantly hampering patient tolerance (9).

Immune checkpoint inhibitors (ICIs), targeting programmed cell death 1 (PD-1) and programmed cell death ligand 1 (PD-L1) has shifted the treatment paradigm for hepatobiliary malignancies. Combining ICIs with standard chemotherapy has emerged as a promising strategy, enhancing clinical outcomes in patients with advanced BTCs. For instance, in the TOPAZ-1 phase 3 trial, patients who received GEMCIS along with durvalumab, a PD-L1 inhibitor, exhibited a statistically significant improvement in OS compared to those treated with GEMCIS and placebo (10). This finding established the combination of GEMCIS with durvalumab as the standard-of-care first-line treatment for advanced BTCs (1, 11). Meanwhile, various real-world studies demonstrated that immunotherapy enhanced the outcomes in patients with iCCA (12–14). Moreover, another phase 3 trial, KEYNOTE-966, a randomized, double-blind, placebo-controlled, multicenter study, examined the combination therapy of pembrolizumab, a PD-1 inhibitor, with systemic chemotherapy. The combination of GEMCIS with pembrolizumab significantly improved the median OS of patients compared to the GEMCIS with the placebo group, with median OS values of 12.7 months vs. 10.9 months, demonstrating its clinical impact in improving patient outcomes (15). Despite these advances, the tolerability and effectiveness of combination therapies are often limited by chemotherapy-related adverse events (AEs), highlighting the need for alternative first-line treatments for advanced iCCA.

Lenvatinib, a multi-targeted tyrosine kinase inhibitor, interferes with multiple oncogenic signaling pathways including vascular endothelial growth factor receptor (VEGFR) 1-3 and fibroblast growth factor receptor (FGFR) 1-4 (16) and has demonstrated its ability to improve therapeutic outcomes across various solid tumors (17). In iCCA preclinical studies, lenvatinib has been proven to exert antitumor effects through cancer cell signaling pathways and the tumor microenvironment (18, 19). A recent phase 2 trial demonstrated that toripalimab plus lenvatinib and GEMOX are promising first-line regimens for the treatment of advanced iCCA. The median OS and PFS were 22.5 and 10.2 months, respectively (20). Preclinical studies have also suggested that lenvatinib may enhance the antitumor effects of PD-1 inhibitor immunotherapy (21, 22). This combination strategy has attracted interest due to its potential to balance efficacy with a more manageable safety profile. However, there is a lack of real-world evidence to support this combination treatment.

Given the therapeutic potential of this treatment, and the need to better understand patient subgroups that would benefit, our study focuses on evaluating the safety and efficacy of Len-P vs. standard systemic chemotherapy (GEMCIS and GEMOX, SC) in patients with iCCA. We aim to identify potential biomarkers for predicting the efficacy of this combination therapy, based on preliminary evidence suggesting that inflammatory markers such as the neutrophil-to-lymphocyte ratio (NLR), lymphocyte-to-C-reactive protein ratio (LCR), lymphocyte-to-monocyte ratio (LMR), systemic immune-inflammation index (SII), and prognostic nutritional index (PNI) may serve as predictive indicators for Len-P treatment response (23).

## Materials and methods

### Patients

**Figure 1:**
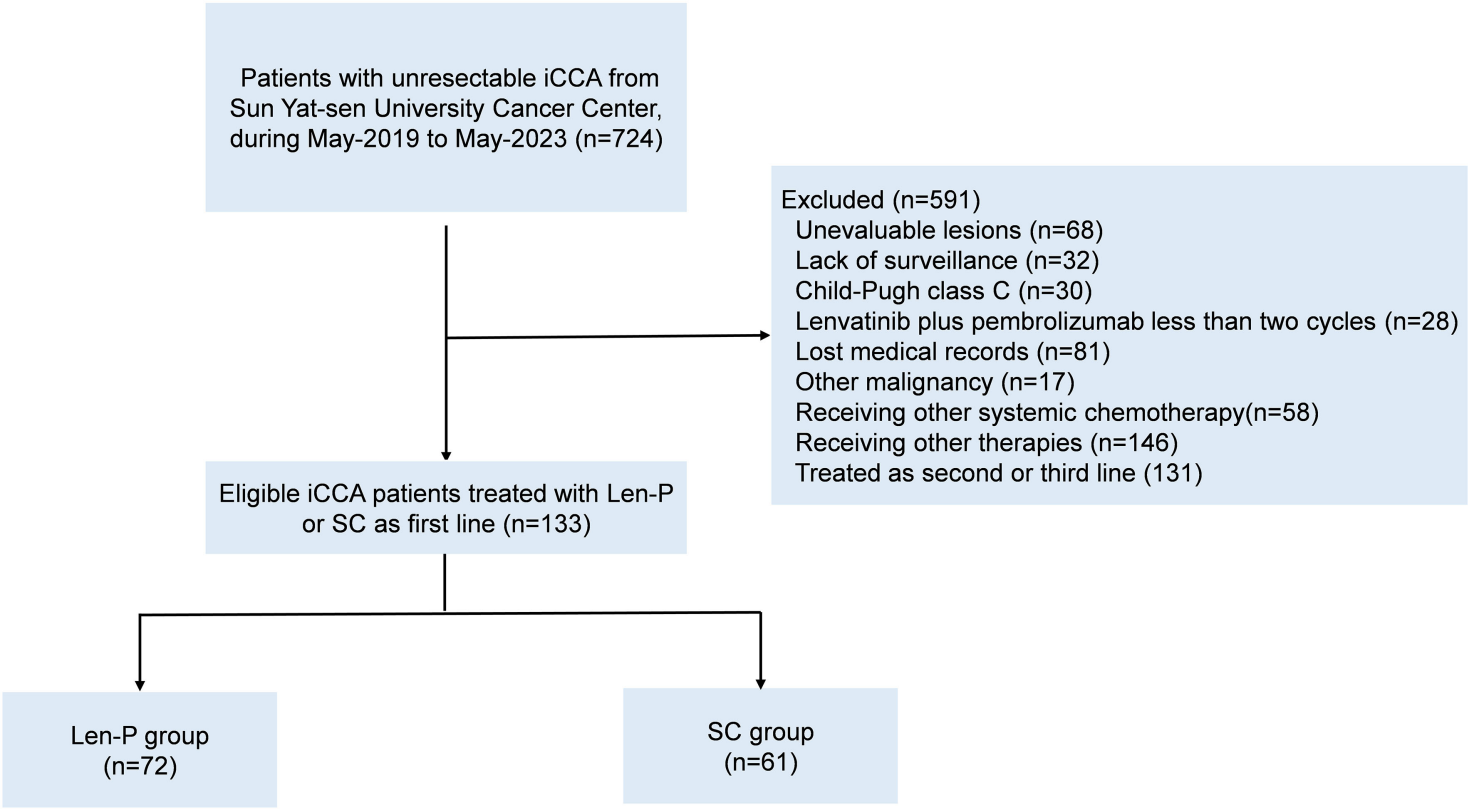
Flowchart for patient inclusion. Abbreviations: iCCA, intrahepatic cholangiocarcinoma; Len-P, lenvatinib-pembrolizumab; SC, systemic chemotherapy.

This retrospective study included a cohort of 133 patients diagnosed with iCCA, who underwent initial treatment with Len-P or first-line SC at Sun Yat-sen University Cancer Center, China, from May 2019 to May 2023. Patients meeting the following criteria were eligible for inclusion: aged 18 years or older; histopathological confirmation of iCCA; initial treatment with Len-P or first-line SC; and an Eastern Cooperative Oncology Group performance status (ECOG PS) score of 2 or lower. Patients were excluded based on the following exclusion criteria: prior or existing malignancies; unassessable lesions before treatment; absence of monitoring; Child-Pugh class C before treatment; less than two cycles of Len-P; missing medical records; alternative systemic chemotherapy; receiving other therapies, and treated as second-line or later. These criteria are shown in **Figure 1**. The retrospective analysis received approval from the Clinical Research Ethics Committee of our cancer center.

### Treatment procedures

Patients were administered a combination regimen of pembrolizumab and lenvatinib. Those with a body weight less than 60 kg received a daily dose of 8 mg of lenvatinib, whereas those weighing 60 kg or more received a daily dose of 12 mg. Pembrolizumab was administered intravenously at a dosage of 200 mg once every three weeks. GEMCIS and GEMOX were used as first-line chemotherapy regimens. In the GEMCIS cohort, each treatment cycle consisted of cisplatin (25 mg per square meter of body surface area (BSA)) followed by gemcitabine (1000 mg per square meter of BSA), administered on days 1 and 8 every three weeks. In the GEMOX cohort, each cycle included oxaliplatin (85 per square meter of BSA) on day 1 and gemcitabine (1000 per square meter of BSA) on days 1 and 8 every three weeks. Treatment with systemic chemotherapy was discontinued at 24 weeks or in the case of disease progression, intolerable adverse effects, or patient preference. The administration and potential cessation due to adverse effects was directed by the drug manufacturer’s prescribing instructions.

### Data collection

All data were sourced from the medical archives of Sun Yat-sen University Cancer Center. Demographic and clinical parameters included: age, gender, ECOG PS, aspartate aminotransferase (AST), alanine transaminase (ALT), albumin (ALB), total bilirubin (TBIL), carbohydrate antigen 19-9 (CA19-9), carcinoembryonic antigen (CEA), white blood cell count (WBC), platelet count (PLT), hemoglobin (HGB), largest tumor size, tumor number, macroscopic vascular invasion, lymph node metastasis, extra-hepatic metastasis, tumor-node-metastasis (TNM) staging, NLR, LCR, LMR, SII, and PNI. The hematological assessments and tumor burdens were determined within 5 days before initial treatment. The NLR, LCR, LMR, SII, and PNI were computed per the methods outlined in **Supplementary Table S1**. Radiographic response was assessed via magnetic resonance imaging (MRI) or computed tomography (CT) conducted at baseline and, after treatment initiation, every 6 weeks. The Response Evaluation Criteria in Solid Tumors (RECIST) 1.1, including complete response (CR), partial response (PR), stable disease (SD), and progressive disease (PD), was used to evaluate tumor response (24).

The primary endpoints in this study were overall survival (OS) and progression-free survival (PFS). OS was defined as the duration spanning from the initiation of treatment to cancer-related death. PFS was characterized as the time from initial treatment to disease advancement, iCCA recurrence, the event of death attributable to iCCA, or the most recent follow-up date. The secondary endpoints encompassed the objective response rate (ORR), disease control rate (DCR), and incidence of treatment-related adverse events (AEs). ORR was specified as the proportion of patients achieving either CR or PR, with a requisite minimum duration of 4 weeks from the initial radiographic verification. DCR was defined as the combination of ORR and the ratio of patients exhibiting SD. Adverse events were evaluated as per CTCAE version 5.0.

### Statistical analysis

Non-normally distributed data were represented asmedians and ranges. Continuous parametric variables were analyzed with the unpaired Student’s t-test, while continuous non-parametric variables were analyzed using the Mann–Whitney U test. Categorical data were evaluated using Pearson’s correlation coefficient chi-square test with continuity correction or Fisher’s exact probability method. Univariate and multivariate Cox regression analyses based on the Forward LR method were executed to identify independent predictive variables. To ensure consistency in the cutoff values of prognostic scores within the cohort, the optimal cutoff point for single value indicators such as NLR, LCR, LMR, SII, and PNI was determined utilizing R version 4.0.1 (**Supplementary Figures S1A–E**). OS and PFS were presented through Kaplan-Meier curves, and inter-group disparities were assessed using the log-rank test results. Statistical significance was at a P value < 0.05. All statistical analyses were carried out using SPSS 25.0 software (SPSS Inc., Chicago, IL) and R version 4.0.1.

## Results

### Patient characteristics

During the period spanning from May 2019 to May 2023, a total of 133 patients with iCCA underwent initial treatment with Len-P or first-line SC were retrospectively enrolled at Sun Yat-sen University Cancer Center in China. Among them, 72 patients were enrolled in the Len-P group, while 61 patients comprised in the SC group, as depicted in **Figure 1**. Detailed demographic characteristics of each group are presented in **Table 1**. There were no significant baseline characteristics observed between the Len-P and SC groups. In the Len-P group, the average age was 55.9 years, with 40 male patients. The mean size of the largest tumor was 7 cm. Most patients presented with multiple tumors (77.8%), 26 individuals (36.1%) exhibited macrovascular invasion, 58 patients (80.6%) had lymph node metastasis, and 30 patients (41.7%) had extra-hepatic metastasis. In the SC group, the mean age was 56.1 years old and 33 patients were male. The mean largest tumor size was 6.9 cm, most patients had multiple tumors (68.9%), 17 patients (27.9%) had macrovascular invasion, 42 patients (68.9%) had lymph node metastasis, and 22 patients (36.1%) had extra-hepatic metastasis. Based on tumor characteristics, the majority of patients in this study presented with a substantial tumor burden and had advanced iCCA. Several systemic inflammatory markers, including NLR, LCR, LMR, SII, and PNI were analyzed. There were no significant differences between the two groups.

**Table 1:** Baseline characteristics of the two group patients.

Variables	Len-P group (n=72)	SC group (n=61)	*P* value
Age(years)	55.9 ± 10.6	56.1 ± 10.1	0.648
Gender(male/female)	40/32 (55.6/44.4)	33/28 (54.1/45.9)	0.866
Hepatitis (yes/no)	55/17 (76.3/23.7)	39/22 (63.9/36.1)	0.116
ECOG PS(1-2/0)	44/28 (61.1/28.9)	37/24 (60.7/39.3)	0.957
ALBI grade (I/II)	57/15 (79.2/20.8)	44/17 (72.1/27.9)	0.344
Preoperative blood tests			
AST, IU/L	47.3 ± 34.4	43.3 ± 30.3	0.421
ALT, IU/L	37.2 ± 27.2	38.6 ± 33.6	0.992
ALB, g/L	42.7 ± 4.3	41.4 ± 3.6	0.167
TBIL, umol/L	17.5 ± 15.4	18.6 ± 32.9	0.725
CEA, ng/ml	3.2 (0.6-1017)	4.6(0.6-8952)	0.223
CA19-9, U/mL	90.1 (2-20000)	152.5 (1.28-20000)	0.657
WBC, 10^9^/L	7.4 ± 2.4	8.5 ± 2.2	0.107
PLT, 10^9^/L	222.7 ± 81.6	263.5 ± 96.6	0.065
HGB, g/L	130.1 ± 22	128.4 ± 16.6	0.723
NLR	3.7 ± 2.8	4.5 ± 2.7	0.068
LCR	4816.7 ± 6532.6	3951.4 ± 9551.1	0.531
LMR	3.7 ± 1.8	3.1 ± 1.6	0.081
SII	867.8 ± 864.8	1122.1 ± 607.4	0.09
PNI	50.9 ± 5.9	49.3 ± 4.8	0.159
Tumor burdens			
Largest tumor size (cm)	7.0 ± 3.7	6.9 ± 3.6	0.756
Tumor number (single/multiple)	16/56 (22.2/77.8)	19/42 (31.1/68.9)	0.244
Macrovascular invasion (yes/no)	26/46 (36.1/63.9)	17/44 (27.9/72.1)	0.311
Lymph node metastasis (yes/no)	58/14 (80.6/19.4)	42/19 (68.9/31.1)	0.119
Extra-hepatic metastasis (yes/no)	30/42 (41.7/58.3)	22/39 (36.1/63.9)	0.51
TNM stage (III-IV /II)	63/9 (87.5/12.5)	49/12 (80.3/19.7)	0.258
Cycles of pembrolizumab	6 (2-20)	–	–
Cycles of chemotherapy	–	2 (2-7)	–

Values are presented as the median (range) or n (%). Categorical variables were compared using Chi-square and Fisher's exact test, continuous parametric variables were analyzed with the unpaired Student's t-test, and continuous non-parametric variables were analyzed using the Mann–Whitney U test.

Len-P, lenvatinib-pembrolizumab; SC, systemic chemotherapy; ECOG PS Eastern Cooperative Oncology Group performance status; ALBI grade, Albumin-Bilirubin grade; AST, aspartate transaminase; ALT, alanine transaminase; ALB, albumin; TBIL, total bilirubin; CEA, carcinoembryonic antigen; CA19-9, carbohydrate antigen 19-9; WBC, white blood cell; PLT, platelet count; HGB, hemoglobin; NLR, neutrophil-to-lymphocyte ratio; LCR, lymphocyte-to-C-reactive protein ratio; LMR, lymphocyte-to-C-reactive protein ratio; SII, systemic immune-inflammation index; PNI, prognostic nutritional index; TNM, tumour-node-metastasis.

### Univariate and multivariable Cox regression analyses

Prognostic factors of all clinical variables were subjected to univariate analysis. The univariate analyses found that ECOG PS, CA19-9, CEA, NLR, LCR, LMR, PNI, macrovascular invasion, and extra-hepatic metastasis were significant risk determinants for OS across all patients. PFS analysis identified ECOG PS, CEA, LCR, macrovascular invasion, and extra-hepatic metastasis as noteworthy risk factors. Further details are outlined in **Table 2**. Subsequent multivariate Cox proportional analysis underscored the significance of CEA (P=0.014), LCR (P<0.001), macrovascular invasion (P=0.001), and extra-hepatic metastasis (P=0.003) as autonomous prognostic indicators of OS (**Table 2**). The multivariate Cox proportional analysis also identified ECOG PS (P=0.001) and CEA (P=0.009) as significant and autonomous prognostic elements for PFS (**Table 2**).

**Table 2:** Univariate and multivariate cox regression analyses of risk factors for overall survival and progression free survival in all patients.

Variables	OS				PFS			
	Univariate		Multivariate		Univariate		Multivariate	
	HR(95% CI)	*P* value	HR(95% CI)	*P* value	HR(95% CI)	*P* value	HR(95% CI)	*P* value
Age, y (>/≤50)	0.97 (0.58-1.63)	0.91			0.77 (0.48-1.24)	0.28		
Gender (male/female)	0.89 (0.55-1.44)	0.64			1 (0.63-1.6)	0.98		
ECOG PS (≥1/0)	1.93 (1.14-3.27)	0.011			2.48 (1.52-4.06)	<0.001	2.31 (1.41-3.8)	0.001
ALBI grade (II/I)	1.36 (0.79-2.36)	0.28			1.28 (0.76-2.16)	0.36		
CA19-9,U/mL, (>/≤100)	1.69 (1.04-2.76)	0.035			1.51 (0.96-2.38)	0.08		
CEA,ng/ml (>/≤5)	2.16 (1.33-3.5)	0.002	1.87 (1.13-3.07)	0.014	2.09 (1.3-3.37)	0.003	1.89 (1.17-3.06)	0.009
NLR (1/0)	1.91 (1.18-3.11)	0.009			1.52 (0.96-2.4)	0.072		
LCR (1/0)	2.16 (1.33-3.5)	0.002	2.97 (1.76-5)	<0.001	1.69 (1.06-2.69)	0.031		
LMR (1/0)	1.78 (1.1-2.91)	0.021			1.55 (0.97-2.47)	0.07		
SII (1/0)	1.5 (0.87-2.58)	0.16			1.65 (0.98-2.78)	0.072		
PNI (1/0)	1.68 (1-2.82)	0.043			1.18 (0.74-1.87)	0.5		
Largest tumor size (>/≤5 cm)	1.64 (0.93-2.88)	0.075			1.2 (0.72-2)	0.48		
Tumor number (>1/1)	0.9 (0.53-1.54)	0.71			0.94 (0.56-1.55)	0.8		
Macrovascular invasion (yes/no)	2.29 (1.39-3.77)	0.002	2.41 (1.42-4.07)	0.001	1.74 (1.09-2.77)	0.023		
Lymph node metastasis (yes/no)	0.85 (0.48-1.53)	0.6			0.84 (0.5-1.4)	0.51		
Extra-hepatic metastasis (yes/no)	2.08 (1.28-3.38)	0.003	2.22 (1.32-3.72)	0.003	1.62 (1.03-2.55)	0.04		
Therapy (SC/Len-P)	1.06 (0.65-1.71)	0.82			0.87 (0.54-1.39)	0.56		

P-value < 0.05 is statistically significant in both univariate and multivariate analyses.

ECOG PS Eastern Cooperative Oncology Group performance status; ALBI grade, Albumin-Bilirubin grade; CEA, carcinoembryonic antigen; CA19-9, carbohydrate antigen 19-9; NLR, neutrophil-to-lymphocyte ratio; LCR, lymphocyte-to-C-reactive protein ratio; LMR, lymphocyte-to-C-reactive protein ratio; SII, systemic immune-inflammation index; PNI, prognostic nutritional index; Len-P, lenvatinib-pembrolizumab; SC, systemic chemotherapy.

### Patient survival and tumor response

**Figure 2:**
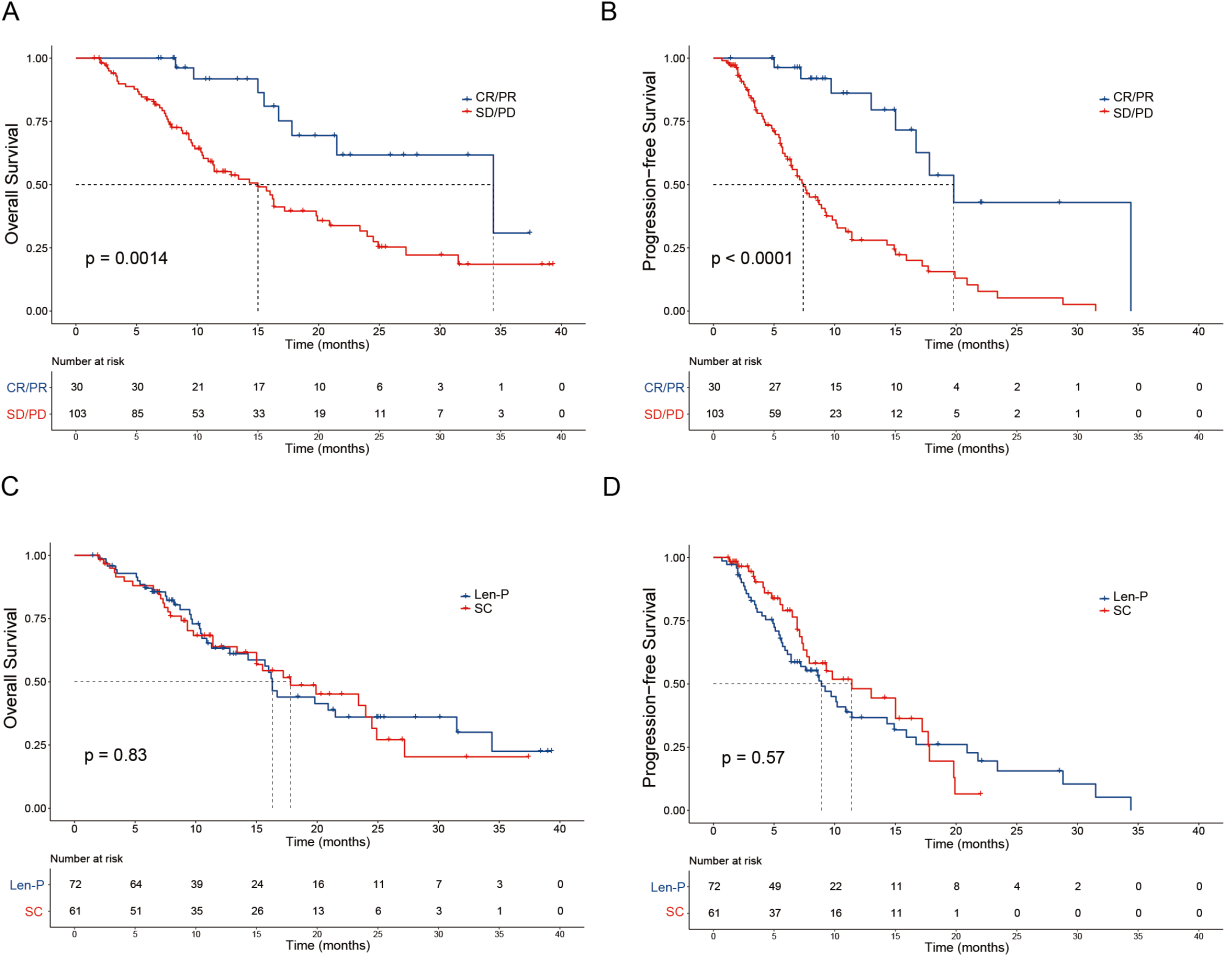
Patient survival was shown by the Kaplan–Meier curves. The overall survival **(A)** and progression-free survival **(B)** in patients with tumor response (CR/PR vs SD/PD) according to RECIST1.1 criteria. The overall survival **(C)** and progression-free survival **(D)** in patients treated with Len-P versus SC. p values were assessed using the log-rank test. RECIST1.1, Response Evaluation Criteria in Solid Tumors 1.1; CR, complete response; PR, partial response; SD, stable disease; PD, progressive disease; Len-P, lenvatinib-pembrolizumab; SC, systemic chemotherapy.

The median OS for the Len-P and SC groups was 16.3 and 17.8 months, respectively. The median PFS for the Len-P and SC groups was 8.9 and 11.4 months, respectively. Noteworthy improvements in OS were seen in patients with positive tumor response (CR and PR) in contrast to non-responders (SD and PD) (P = 0.0014; **Figure 2A**). Furthermore, responders (CR and PR) had a prolonged PFS compared to non-responders (SD and PD) (P < 0.0001; **Figure 2B**). No significant differences were observed between the Len-P and SC groups concerning OS (P=0.83; **Figure 2C**) and PFS (P=0.57; **Figure 2D**). The median follow-up duration for the Len-P group and SC group was 22.6 months and 19.7 months, respectively. Patients in the SC group were stratified into two subgroups (GEMCIS and GEMOX). Comparative analyses of GEMCIS and GEMOX against Len-P were conducted in regard to OS and PFS (**Supplementary Figure S2**).

**Figure 3:**
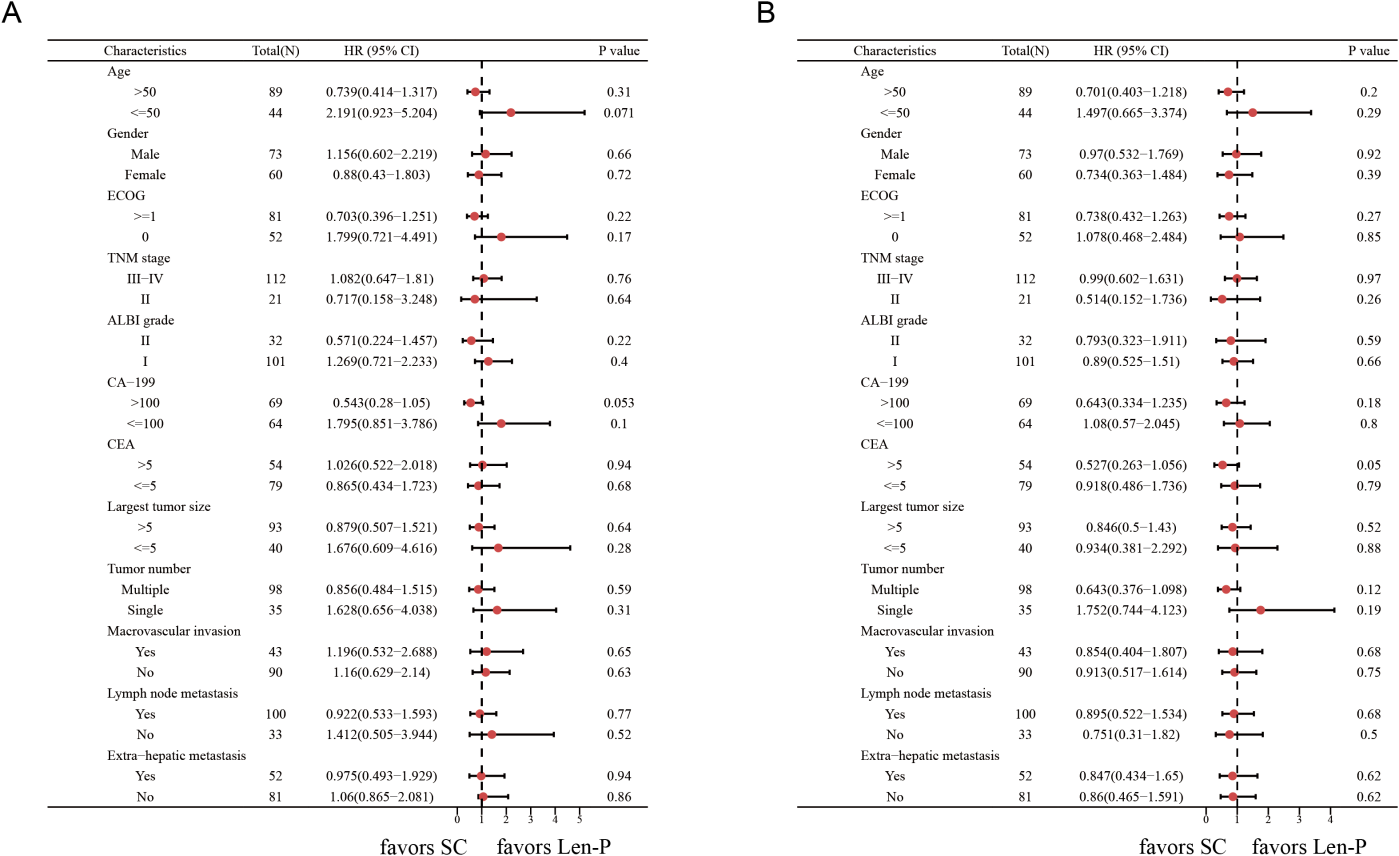
Forest plots of **(A)** overall survival and **(B)** progression-free survival in different patient subgroups. p values were assessed using the log-rank test. HR, hazard ratio; CI, confidence interval; ECOG, hepatic arterial infusion chemotherapy; TNM, tumor-node-metastasis; ALBI grade, Albumin-Bilirubin grade; CA19-9, carbohydrate antigen 19-9; CEA, carcinoembryonic antigen. Len-P, lenvatinib-pembrolizumab; SC, systemic chemotherapy.

**Figure 4:**
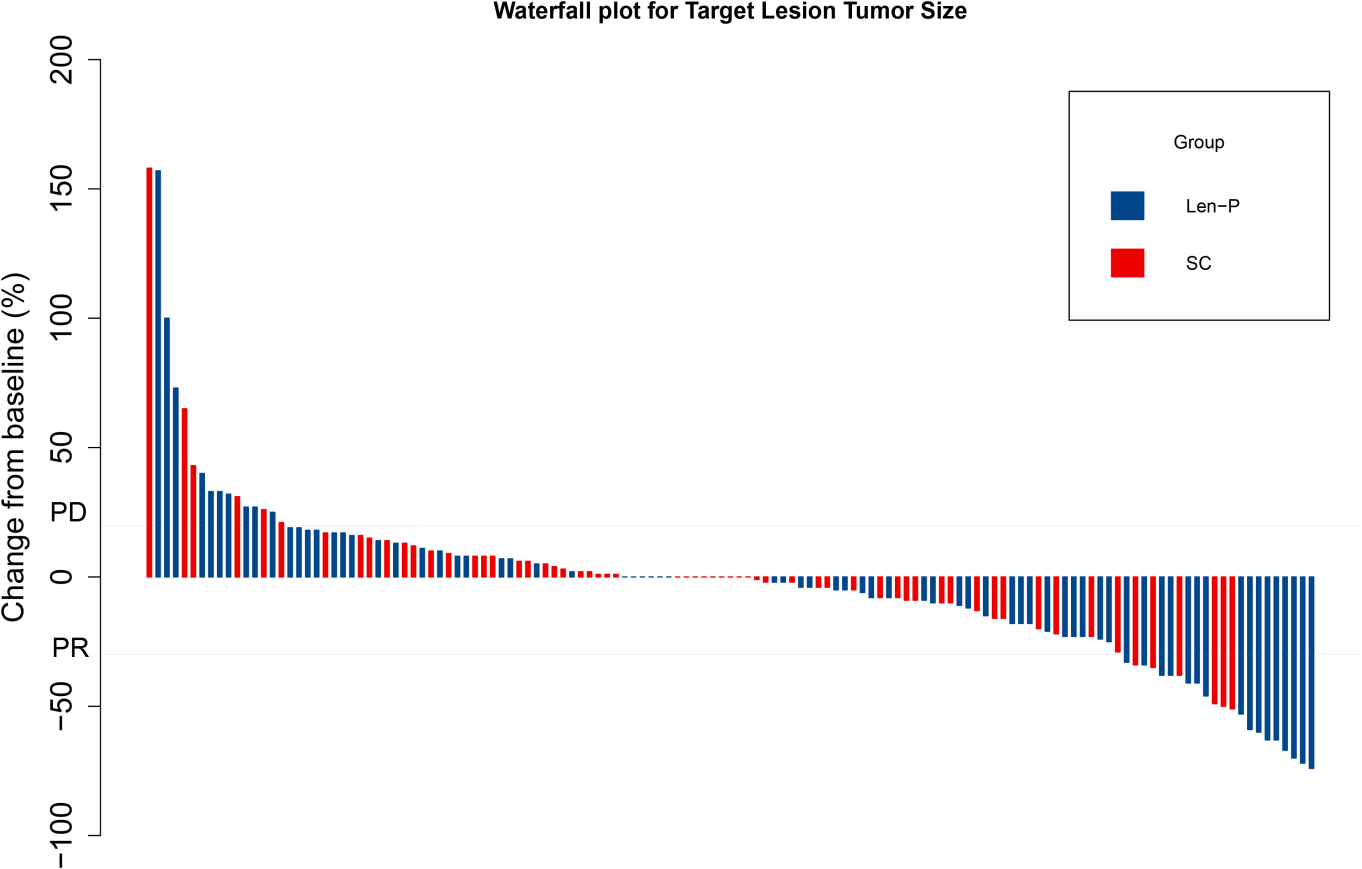
Waterfall plot for tumor size changes of intra-hepatic target lesions. PD, progressive disease; PR, partial response. Len-P, lenvatinib-pembrolizumab; SC, systemic chemotherapy.

The subgroup analyses for OS and PFS are presented in **Figures 3A, B**. Len-P conferred comparable clinical benefit in terms of both OS and PFS across the various subgroups when compared to SC. The tumor responses of the patients are detailed in **Table 3**. According to RECIST 1.1 criteria, there was no significant difference in ORR and DCR between the Len-P and SC group (ORR: 22.2% vs 23%; P=0.92; DCR: 69.4% vs 77%; P=0.58). The optimal response for intra-hepatic target lesions by RECIST1.1 criteria is illustrated in the waterfall plot in **Figure 4**.

**Table 3:** Tumor responses evaluated by RECIST1.1 criteria.

Response	RECIST1.1		
	Len-P group (n=72)	SC group (n=61)	*P* value
CR	0	0	–
PR	16 (22.2%)	14 (23%)	–
SD	34 (47.2%)	33 (54%)	–
PD	22 (30.6%)	14 (23%)	–
ORR	16 (22.2%)	14 (23%)	0.92
DCR	55 (69.4%)	49 (77%)	0.58

Categorical variables were compared using Chi-square test.

Len-P, lenvatinib-pembrolizumab; SC, systemic chemotherapy; CR, complete response; PR, partial response; SD, stable disease; PD, progressive disease; ORR, objective response rate; DCR, disease control rate.

### Safety

SC led to a higher frequency of AEs in comparison to Len-P, as shown in **Table 4**. The following AEs exhibited lower prevalence in the Len-P compared to SC group: abdominal pain (5 (6.9%) vs. 14 (22.9%); P=0.009), vomiting (6 (8.3%) vs. 28 (45.9%); P<0.001), fatigue (10 (13.9%) vs. 24 (39.3%); P=0.001), leukopenia (8 (11.1%) vs. 15 (24.6%); P=0.041), anemia (10 (13.9%) vs. 26 (42.6%); P<0.001), thrombocytopenia (8 (11.1%) vs. 16 (26.2%); P=0.024), and sensory neuropathy (9 (12.5%) vs. 16 (26.2%); P=0.043). A notable difference was found in the overall occurrence of severe AEs, which was higher in the SC group than in the Len-P group. In the Len-P cohort, treatment discontinuation due to adverse events occurred in 4 patients (5.6%), while in the SC cohort, this number was 8 patients (13.1%). All adverse events were effectively managed, and no mortality attributable to treatment toxicity was reported in the follow-up period.

**Table 4:** Objective treatment-related adverse events.

Adverse Events	Any Grade			Grade3-4		
	Len-P group (n=72)	SC group (n=61)	*P* value	Len-P group (n=72)	SC group (n=61)	*P* value
Rash	13 (18.1%)	10 (16.4%)	0.801	1	0	1.000
Fever	8 (11.1%)	13 (21.3%)	0.116	0	0	**-**
Abdominal pain	5 (6.9%)	14 (22.9%)	0.009	0	0	**-**
Vomiting	6 (8.3%)	28 (45.9%)	<0.001	1 (1.3%)	8 (13.1%)	0.019
Fatigue	10 (13.9%)	24 (39.3%)	0.001	0	0	**-**
Diarrhea	9 (12.5%)	10 (16.4%)	0.523	0	0	**-**
Leukopenia	8 (11.1%)	15 (24.6%)	0.041	0	3 (4.9%)	0.094
Neutropenia	3 (4.2%)	9 (14.8%)	0.069	0	2 (3.3%)	0.208
Anemia	10 (13.9%)	26 (42.6%)	<0.001	1 (1.3%)	5 (8.2%)	0.143
Thrombocytopenia	8 (11.1%)	16 (26.2%)	0.024	0	2 (3.3%)	0.208
Elevated ALT	10 (13.9%)	9 (14.8%)	0.887	0	0	**-**
Elevated AST	13 (18.1%)	10 (16.4%)	0.801	0	0	**-**
Hyperbilirubinemia	12 (16.7%)	9 (14.8%)	0.763	0	0	**-**
Hypoalbuminemia	28 (38.9%)	26 (42.6%)	0.662	0	1 (1.6%)	0.459
Elevated creatinine	9 (12.5%)	13 (21.3%)	0.173	0	0	**-**
Sensory neuropathy	9 (12.5%)	16 (26.2%)	0.043	0	0	**-**

Some patients may have multiple immune-related adverse events. Categorical variables were compared using Chi-square and Fisher's exact test.

Len-P, lenvatinib-pembrolizumab; SC, systemic chemotherapy; ALT, alanine aminotransferase; AST, aspartate aminotransferase.

### Inflammation-based scores analysis for Len-P group

In the Len-P group, the univariate analyses revealed that inflammation-based scores, including NLR, LCR, LMR, SII, and PNI emerged as significant risk factors impacting OS. The multivariate Cox proportional analysis particularly emphasized the roles of NLR (P=0.045) and LMR (P=0.011) as independent and significant prognostic determinants for OS (**Supplementary Table S2**). All inflammation-based scores were associated with OS outcomes of patients undergoing Len-P treatment. Specifically, low NLR (P=0.0021), LCR (P=0.00018), LMR (P=0.00026), SII (P=0.006), and PNI (P=0.028) scores were indicative of favorable prognoses (**Supplementary Figures S3A–E**).

## Discussion

The application of pembrolizumab in conjunction with lenvatinib for iCCA has not been thoroughly evaluated within a real-world context previously. In this study, we scrutinized the application of this therapeutic modality in a retrospective real-world cohort, with a specific emphasis on efficacy and safety relative to first-line SC, and identifying patients who may experience an enhanced benefit. We validated the efficacy and safety of the combined therapy as a viable option for patients with advanced iCCA. The strengths of this current study lie in (1) the incorporation of an expanded real-world study cohort in China comprising a total of 133 patients (Len-P vs. SC: 72 vs 61), (2) the implementation of comprehensive outcome subgroup analyses to identify subpopulations that may benefit from screening, and (3) the documentation of long- and short-term treatment outcomes for patients with advanced iCCA undergoing either Len-P or standard chemotherapy.

iCCA is a gastrointestinal adenocarcinoma characterized by a high degree of malignancy and a poor prognosis. Most iCCA patients are ineligible for surgical intervention due to the advanced stage of the disease, leading to the administration of SC to manage tumor progression. In recent years, GEMCIS and GEMOX have emerged as the established first-line chemotherapy regimens (6, 25, 26). Nevertheless, the presence of adverse events presents a challenge for the use of SC. There is a need for a treatment regimen that reduces the occurrence of adverse events while attaining similar survival outcomes.

In the current study of 133 patients, we compared Len-P with first-line SC (GEMCIS and GEMOX) and found that the median OS for the Len-P and SC groups was 16.3 and 17.8 months, respectively (P=0.83), and the median PFS was 8.9 and 11.4 months, respectively (P=0.57). According to RECIST 1.1 criteria, there was no significant difference in ORR and DCR between the Len-P and SC group (ORR: 22.2% vs. 23%; P=0.92; DCR: 69.4% vs. 77%; P=0.58). These findings indicate that Len-P exhibited comparable clinical efficacy to SC in patient outcomes.

Our efficacy data aligns with previous research. In a phase II study of toripalimab combined with lenvatinib as first-line therapy for 31 patients with advanced iCCA, the median OS and PFS were recorded at 22.5 months and 10.2 months, respectively (20). KEYNOTE-966 investigated pembrolizumab in combination with GEMCIS for BTC patients, and found a median OS of 12.7 months (95% CI 11.5–13.6) in the pembrolizumab cohort (15). Furthermore, the phase II LEAP-005 trial evaluated lenvatinib plus pembrolizumab as a second-line regimen for 31 advanced BTC patients, and found a median PFS of 6.1 months, median OS of 8.6 months, ORR of 10%, and DCR of 68% (27). Together, these results provide robust backing for the effectiveness of Len-P in treating advanced iCCA. Len-P is a viable first-line treatment option, especially for patients who cannot tolerate or refuse chemotherapy. To further support our conclusions, prospective randomized controlled clinical trials are needed.

Safety serves as a crucial benchmark in assessing a treatment regimen beyond therapeutic efficacy. Generally, there was a lower incidence of AEs in the Len-P cohort compared to the SC cohort. Abdominal pain, vomiting, fatigue, leukopenia, anemia, thrombocytopenia, and sensory neuropathy were less frequent in the Len-P cohort. Likewise, the prevalence of grade 3–4 adverse events was diminished in the Len-P cohort. In SC cohort, to achieve the desired effect of tumor eradication, the concentration of the drug may reach a level that induces damage to body systems, resulting in AEs. These AEs are typically severe and effective management is challenging. Thus, Len-P emerges as a potentially safe and efficacious therapeutic regimen for patients with advanced iCCA.

To identify the population of patients with the greatest benefit from Len-P, we conducted univariate and multivariable Cox regression analyses. Our study revealed that inflammation-based scores were reliable indicators for predicting the effectiveness of Len-P in patients with iCCA. The etiology of iCCA overlaps with that of primary sclerosing cholangitis, primary biliary cirrhosis, and other conditions characterized by biliary tract inflammation and fibrosis (28). Inflammation plays a pivotal role in the initiation and progression of iCCA (29). Mounting evidence indicates a correlation between the inflammatory environment and response to PD-1 inhibitors in advanced malignancies, including iCCA (23, 30–35). Inflammatory markers could have utility in identifying individuals who are likely to respond better to Len-P therapy.

This study has some limitations. Firstly, the retrospective approach exposes the study to potential selection biases. Its retrospective nature limits us to performing statistical analyses to compare for statistical differences (i.e., P < 0.05), and ultimately, we still need to interpret the significance in conjunction with the clinical context. Therefore, a prospective, multicenter, randomized controlled trial is essential to corroborate our findings. Secondly, the retrospective methodology may have led to an incomplete evaluation of adverse occurrences, despite our thorough examination of the clinical records. Thirdly, the execution of multiple subgroup analyses may have led to a reduction in the sample size and reduce statistical power, necessitating cautious interpretation of the conclusions. Finally, further laboratory studies are warranted to elucidate the fundamental mechanisms underpinning the efficacy of Len-P in patients with iCCA.

## Conclusion

This study demonstrates that Len-P is a promising and safe therapeutic modality for patients with advanced iCCA. It is a viable treatment alternative to SC for advanced iCCA. Inflammatory-based scores have potential utility in identifying individuals more likely to respond well to Len-P therapy.

## Data Availability

The original contributions presented in the study are included in the article/**Supplementary Material**. Further inquiries can be directed to the corresponding authors.

## Supplementary material

Supplementary Figure 1 Schematic of the method used to determine the optimal cutoff points of NLR, LCR, LMR, SII, and PNI using R version for survival prediction. NLR, neutrophil-to-lymphocyte ratio; LCR, lymphocyte-to-C-reactive protein ratio; LMR, lymphocyte-to-C-reactive protein ratio; SII, systemic immune-inflammation index; PNI, prognostic nutritional index.

Supplementary Figure 2 The overall survival and progression-free survival of the two groups of patients. Kaplan-Meier curves of **(A)** overall survival and **(B)** progression-free survival for patients in the Len-P, GEMCIS and GEMOX groups. p values were assessed using the log-rank test. Len-P, lenvatinib-pembrolizumab; GEMCIS, gemcitabine plus cisplatin; GEMOX, oxaliplatin plus gemcitabine.

Supplementary Figure 3 Kaplan–Meier curves of the overall survival of iCCA patients after Len-P therapy. **(A)** NLR **(B)** LCR, **(C)** LMR, **(D)** SII, and **(E)** PNI. p values were assessed using the log-rank test. iCCA, intrahepatic cholangiocarcinoma; NLR, neutrophil-to-lymphocyte ratio; LCR, lymphocyte-to-C-reactive protein ratio; LMR, lymphocyte-to-C-reactive protein ratio; SII, systemic immune-inflammation index; PNI, prognostic nutritional index.
