# Supplementary figures and images for: Comparison of lenvatinib plus pembrolizumab versus first-line systemic chemotherapy for advanced intrahepatic cholangiocarcinoma: a real-world retrospective study
Source: Front Immunol. 2024 Nov 29;15:1494520. doi: 10.3389/fimmu.2024.1494520 (PMC11638178; doi:10.3389/fimmu.2024.1494520)

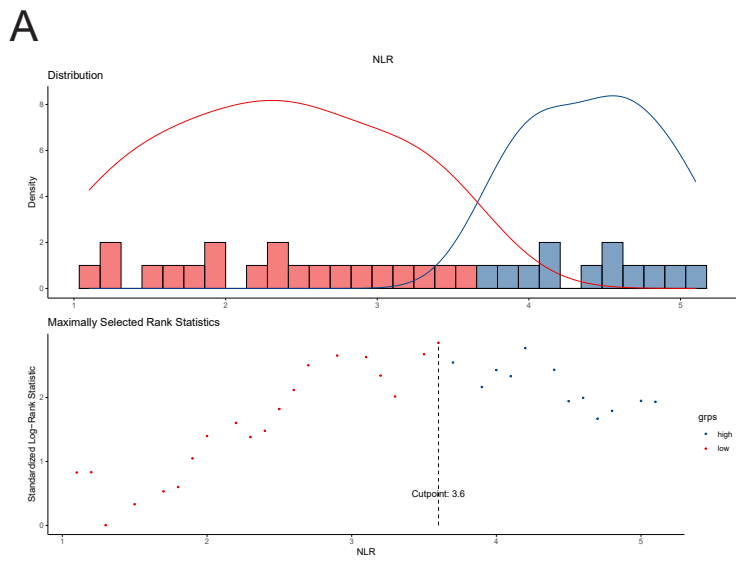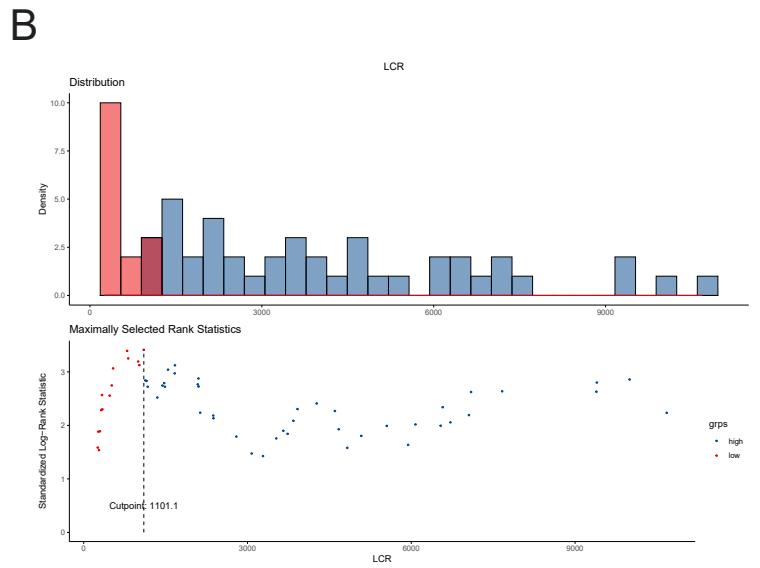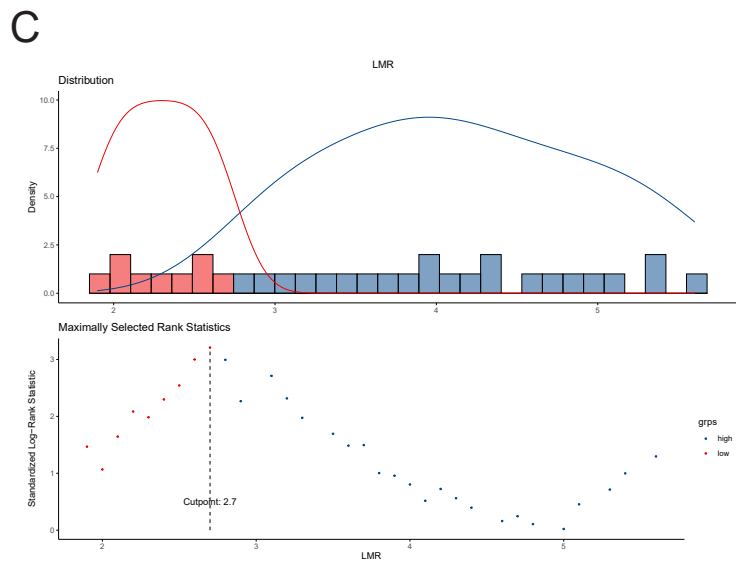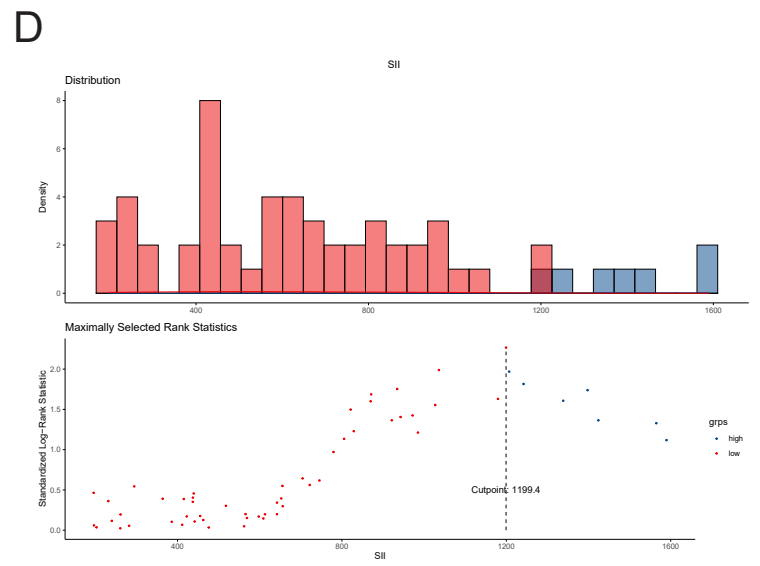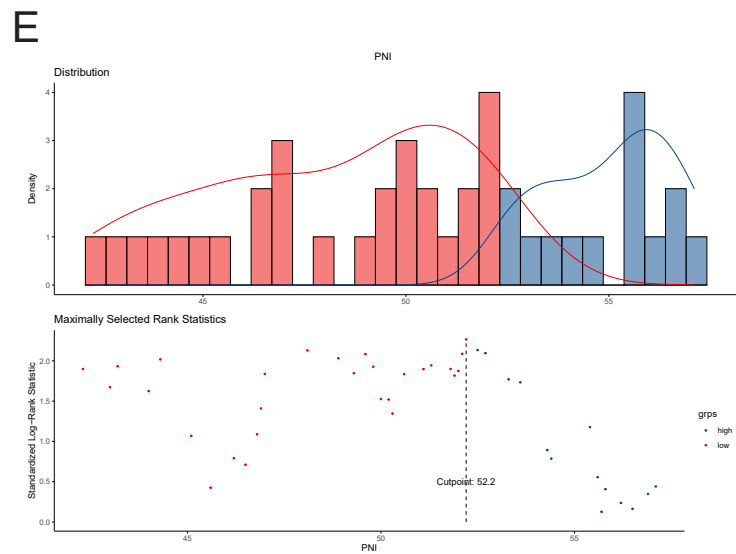

Supplement: Supplementary Figure 1 — Schematic of the method used to determine the optimal cutoff points of NLR, LCR, LMR, SII, and PNI using R version for survival prediction. NLR, neutrophil-to-lymphocyte ratio; LCR, lymphocyte-to-C-reactive protein ratio; LMR, lymphocyte-to-C-reactive protein ratio; SII, systemic immune-inflammation index; PNI, prognostic nutritional index. [file Image1.pdf]

A

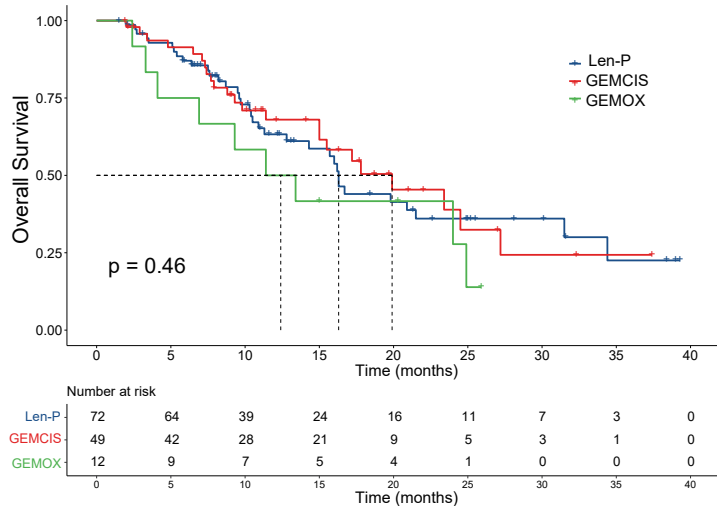

B

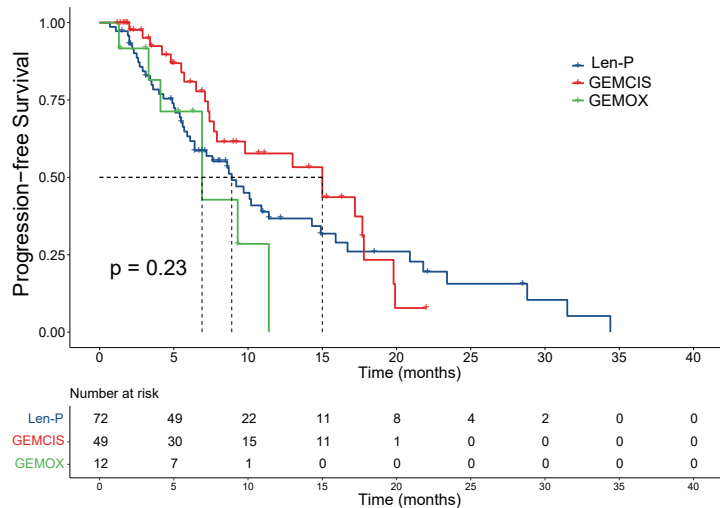

Supplement: Supplementary Figure 2 — The overall survival and progression-free survival of the two groups of patients. Kaplan-Meier curves of (A) overall survival and (B) progression-free survival for patients in the Len-P, GEMCIS and GEMOX groups. p values were assessed using the log-rank test. Len-P, lenvatinib-pembrolizumab; GEMCIS, gemcitabine plus cisplatin; GEMOX, oxaliplatin plus gemcitabine. [file Image2.pdf]

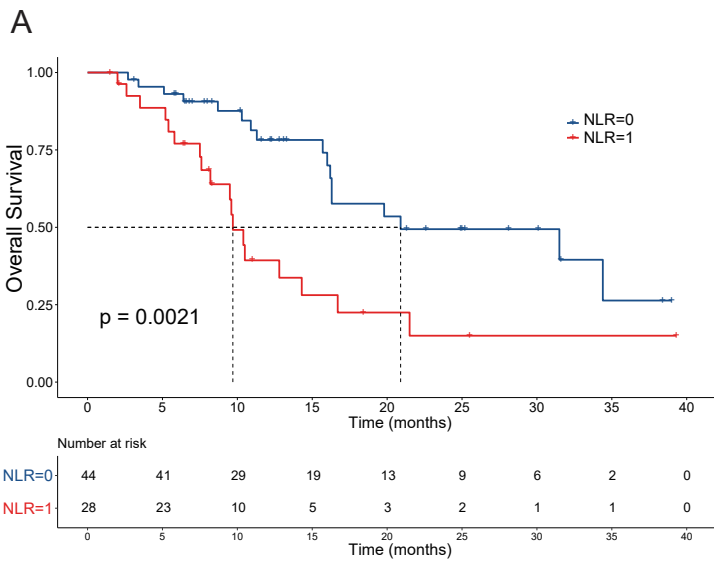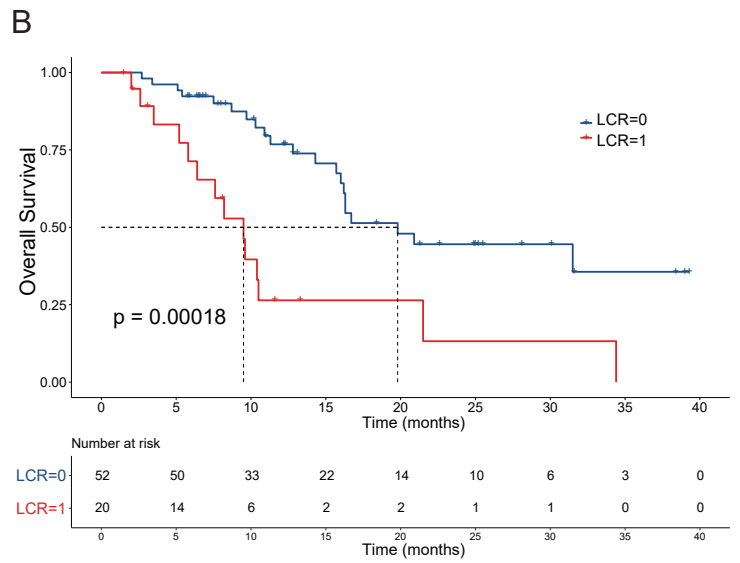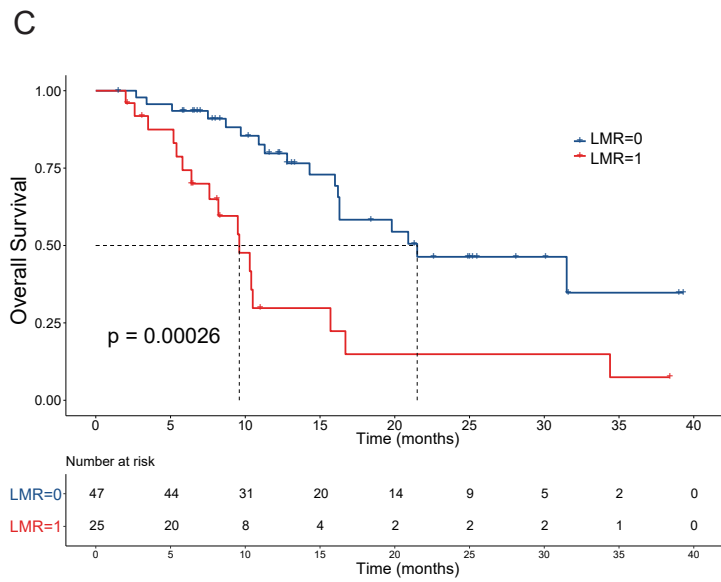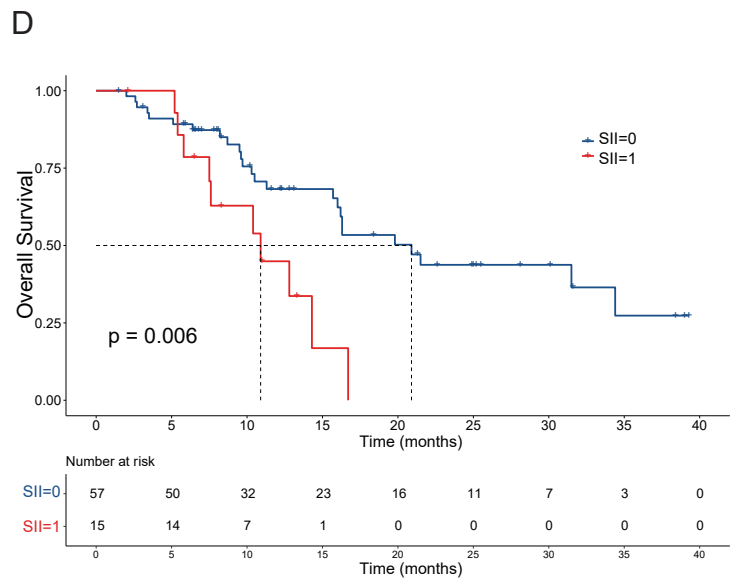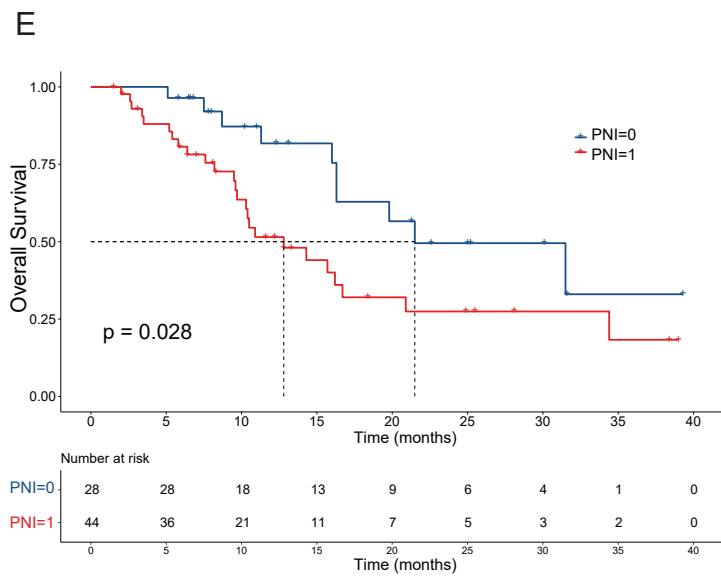

Supplement: Supplementary Figure 3 — Kaplan–Meier curves of the overall survival of iCCA patients after Len-P therapy. (A) NLR (B) LCR, (C) LMR, (D) SII, and (E) PNI. p values were assessed using the log-rank test. iCCA, intrahepatic cholangiocarcinoma; NLR, neutrophil-to-lymphocyte ratio; LCR, lymphocyte-to-C-reactive protein ratio; LMR, lymphocyte-to-C-reactive protein ratio; SII, systemic immune-inflammation index; PNI, prognostic nutritional index. [file Image3.pdf]
