# Supplementary material for: Comparison of lenvatinib plus pembrolizumab versus first-line systemic chemotherapy for advanced intrahepatic cholangiocarcinoma: a real-world retrospective study
Source: Front Immunol. 2024 Nov 29;15:1494520. doi: 10.3389/fimmu.2024.1494520 (PMC11638178; doi:10.3389/fimmu.2024.1494520)
Supplement: Supplementary file 4 [file Table1.docx]

**Table S1: Systemic Inflammation-Based Prognostic Scores**

| **Scoring System** | **Score** |
| --- | --- |
| Neutrophil to lymphocyte ratio (NLR) |  |
| Neutrophil count (×109/L): lymphocyte count (×109/L) ≤ 3.6 | 0 |
| Neutrophil count (×109/L): lymphocyte count (×109/L) > 3.6 | 1 |
| Lymphocyte to C-reactive protein ratio (LCR) |  |
| 10^4^×lymphocyte count (×109/L): CRP (mg/L) > 1101.1 | 0 |
| 10^4^×lymphocyte count (×109/L): CRP (mg/L) ≤ 1101.11 | 1 |
| Lymphocyte to monocyte ratio (LMR) |  |
| Lymphocyte count (×109/L): monocyte count (×109/L) > 2.7 | 0 |
| Lymphocyte count (×109/L): monocyte count (×109/L) ≤ 2.7 | 1 |
| Systemic Immune-inflammation Index (SII) |  |
| Platelet count (×109/L) × neutrophil count (×109/L)/lymphocyte count (×109/L) ≤ 1199.4 | 0 |
| Platelet count (×109/L) × neutrophil count (×109/L)/lymphocyte count (×109/L) > 1199.4 | 1 |
| Prognostic Nutritional Index (PNI) |  |
| Albumin (g/L) + 5 × lymphocyte count (×109/L) > 52.2 | 0 |
| Albumin (g/L) + 5 × lymphocyte count (×109/L) ≤ 52.2 | 1 |

Abbreviations: CRP, C-reactive protein
