# Supplementary material for: Comparison of lenvatinib plus pembrolizumab versus first-line systemic chemotherapy for advanced intrahepatic cholangiocarcinoma: a real-world retrospective study
Source: Front Immunol. 2024 Nov 29;15:1494520. doi: 10.3389/fimmu.2024.1494520 (PMC11638178; doi:10.3389/fimmu.2024.1494520)
Supplement: Supplementary file 5 [file Table2.docx]

**Table 2: Univariate and Multivariate Cox Regression Analyses of Risk Factors for Overall Survival and Progression Free Survival in Len-P group.**

| **Variables** | **OS** | | | | **PFS** | | | |
| --- | --- | --- | --- | --- | --- | --- | --- | --- |
|  | **Univariate** | | **Multivariate** | | **Univariate** | | **Multivariate** | |
|  | **HR(95% CI)** | ***P* value** | **HR(95% CI)** | ***P* value** | **HR(95% CI)** | ***P* value** | **HR(95% CI)** | ***P* value** |
| Age, y (>/≤50) | 1.68 (0.76-3.72） | 0.18 |  |  | 1.09 (0.59-2) | 0.79 |  |  |
| Gender (male/female) | 0.79 (0.4-1.57) | 0.51 |  |  | 0.92 (0.51-1.68) | 0.79 |  |  |
| ECOG PS (≥1/0) | 3.04 (1.44-6.41) | 0.004 | 5.49 (2.21-13.65) | <0.001 | 2.81 (1.5-5.29) | 0.001 | 3.67 (1.87-7.19) | <0.001 |
| ALBI grade (II/I) | 2.21 (1.05-4.67) | 0.038 | 3.71 (1.38-9.95) | 0.009 | 1.34 (0.68-2.65) | 0.4 |  |  |
| CA19-9,U/mL, (>/≤100) | 3.06 (1.51-6.21) | 0.002 |  |  | 1.71 (0.95-3.07) | 0.073 |  |  |
| CEA,ng/ml (>/≤5) | 1.92 (0.98-3.76) | 0.057 |  |  | 2.49 (1.33-4.68) | 0.005 |  |  |
| NLR (1/0) | 2.75 (1.4-5.38) | 0.003 | 2.16 (1.02-4.57) | 0.045 | 1.92 (1.07-3.44) | 0.03 |  |  |
| LCR (1/0) | 3.46 (1.73-6.9) | <0.001 |  |  | 1.72 (0.91-3.25) | 0.095 |  |  |
| LMR (1/0) | 3.34 (1.69-6.62) | 0.001 | 3.13 (1.3-7.56) | 0.011 | 2.03 (1.11-3.71) | 0.021 | 2.94 (1.52-5.67) | 0.001 |
| SII (1/0) | 2.86 (1.31-6.26) | 0.009 |  |  | 1.87 (0.93-3.76) | 0.081 |  |  |
| PNI (1/0) | 2.24 (1.07-4.68) | 0.033 |  |  | 1.01 (0.56-1.81) | 0.97 |  |  |
| Largest tumor size (>/≤5 cm) | 2.05 (0.93-4.52) | 0.075 |  |  | 1.33 (0.71-2.53) | 0.38 |  |  |
| Tumor number (>1/1) | 1.39 (0.57-3.36) | 0.47 |  |  | 1.46 (0.68-3.13) | 0.34 |  |  |
| Macrovascular invasion (yes/no) | 2.27 (1.15-4.48) | 0.018 |  |  | 1.69 (0.94-3.04) | 0.078 |  |  |
| Lymph node metastasis (yes/no) | 1.24 (0.47-3.25) | 0.66 |  |  | 0.73 (0.36-1.48) | 0.39 |  |  |
| Extra-hepatic metastasis (yes/no) | 2.13 (1.08-4.18) | 0.028 | 3.43 (1.61-7.33) | 0.001 | 1.54 (0.86-2.77) | 0.15 |  |  |

**Note:** P-value < 0.05 is statistically significant in both univariate and multivariate analyses

**Abbreviations:** Len-P, lenvatinib-pembrolizumab; ECOG PS Eastern Cooperative Oncology Group performance status; ALBI grade, Albumin-Bilirubin grade; CEA, carcinoembryonic antigen; CA19-9, carbohydrate antigen 19-9; NLR, neutrophil-to-lymphocyte ratio; LCR, lymphocyte-to-C-reactive protein ratio; LMR, lymphocyte-to-C-reactive protein ratio; SII, systemic immune-inflammation index; PNI, prognostic nutritional index.
